# H5N1 clade 2.3.2.1e lineage transition in Lao PDR during 2023–2024 associated with regional spread and human infection risk-associated receptor-binding features

**DOI:** 10.3389/fpubh.2026.1923867

**Published:** 2026-09-03

**Authors:** Duc Duong Than, Boyoon Chang, Eun Lee, Watthana Theppangna, Seon-Ju Yeo, Hyun Park

**Affiliations:** 1 Zoonosis Research Center, Department of Infection Biology, School of Medicine, Wonkwang University, Iksan, Republic of Korea; 2 Institute of Endemic Diseases, Seoul National University Medical Research Center, Seoul, Republic of Korea; 3 National Animal Health Laboratory, Vientiane, Laos; 4 Department of Tropical Medicine and Parasitology, Department of Biomedical Sciences, College of Medicine, Seoul National University, Seoul, Republic of Korea

**Keywords:** Lao PDR, influenza A(H5N1), clade 2.3.2.1e, genetic reservoir, mammalian adaptation

## Abstract

Highly pathogenic avian influenza (HPAI) H5N1 viruses clade 2.3.2.1e has re-emerged in the Indochinese Peninsula, raising concerns about regional spread and zoonotic risk. We characterized eight H5N1 viruses isolated from 32 environmental and poultry-derived samples collected in Lao People’s Democratic Republic (Lao PDR) live bird markets during 2023–2024. Whole-genome and phylogenetic analyses showed that all isolates belonged to clade 2.3.2.1e and were distinct from the previously dominant Lao clade 2.3.2.1c, indicating lineage transition in poultry-associated viruses. The Lao isolates were distributed across regional sublineages rather than forming a single local cluster, suggesting repeated introductions and cross-border genetic connectivity. Segment-level nucleotide identities ranged from 97.2 to 99.9%, revealing a complex mosaic genotype constellation. All isolates retained the avian-associated glutamic acid residue at position 627 of polymerase basic protein 2 (PB2-627E) and the aspartic acid residue at position 701 of polymerase basic protein 2 (PB2-701D). Hemagglutinin (HA) analysis showed a conserved polybasic cleavage motif, PQRERRRKRGLF, and conserved HA1-123S, HA1-154N, HA1-263T, and HA2-497L, whereas isolate-level variation was observed at HA1-94, HA1-188, and HA1-223. Structural modeling and Molecular Mechanics-Generalized Born Surface Area (MM-GBSA) analysis of a representative isolate further highlighted receptor binding site (RBS) associated HA features. Importantly, RBS-associated HA variation was detected despite the absence of lysine residue at position 627 of polymerase basic protein 2 (PB2-627 K), suggesting that early molecular changes relevant to mammalian or human adaptation may arise before acquisition of canonical mammalian-adaptive polymerase mutations. These findings support sustained cross-border genomic surveillance and receptor-binding-focused characterization of H5N1 viruses circulating in Lao live bird markets.

## 1 Introduction

Highly pathogenic avian influenza (HPAI) H5N1 viruses of the A/goose/Guangdong/1/96 lineage continue to pose a substantial threat to both animal health and public health (1, 2). Since their emergence, these viruses have diversified into multiple clades with distinct geographic distributions, evolutionary trajectories, and host adaptation profiles (3, 4). In Southeast Asia, the Indochinese Peninsula is an important setting for H5N1 persistence and spread. This is driven by dense poultry production, widespread live bird market (LBM) systems, and frequent cross-border movement of birds and poultry products. Recent market-based surveillance in Laos has detected avian influenza viruses in live animal and wet market environments, further emphasizing the relevance of market-based surveillance in this region (5).

Within this regional context, the Lao People’s Democratic Republic (Lao PDR) occupies a strategically important position. Sharing borders with China, Vietnam, Thailand, Cambodia, and Myanmar, Lao PDR lies at the intersection of several poultry trade routes and migratory pathways that may facilitate repeated viral incursions and onward dissemination (6). Previous surveillance has shown that Lao PDR has experienced multiple introductions of HPAI H5 viruses, including clades 1, 2.3.4, 2.3.2, and 2.3.4.4, with clade 2.3.2.1c becoming one of the major lineages detected in the country and neighboring areas during earlier periods of circulation (3). These observations suggest that Lao PDR is not merely a recipient of imported viruses, but a setting in which H5 lineages may persist, diversify, and contribute to broader regional dynamics.

Recent events in the lower Mekong region have elevated concerns regarding the zoonotic potential of emerging H5N1 lineages. Between 2023 and 2024, clade 2.3.2.1e (formerly 2.3.2.1c) re-emerged in the Indochinese Peninsula, associated with high mortality in both humans and wildlife. Cambodia reported a fatal resurgence of human infections starting in early 2023, with a case fatality rate ranging between 38 and 44.4% (2, 7). Simultaneously, in late 2024, Vietnam witnessed an unprecedented outbreak in captive felids, where 47 tigers and 3 leopards died rapidly from clade 2.3.2.1e infection (8). Genomic characterization of these mammalian cases revealed the fixation of critical host-adaptation markers, most notably the polymerase basic protein 2 (PB2) E627K mutation, which enhances viral polymerase activity and replication in mammalian cells (9, 10).

Despite these regional developments, the genetic characteristics of H5N1 viruses circulating in Lao PDR remain insufficiently resolved. Although recent surveillance has documented increased avian influenza virus positivity in Lao live animal and wet market environments and detected HPAI H5N1 clade 2.3.2.1e, detailed whole-genome data for currently circulating Lao strains remain limited (5). It therefore remains unclear how Lao avian reservoir viruses are genetically related to highly virulent mammalian-associated strains reported in neighboring countries, including recent human infections in Cambodia and the tiger/leopard mortality event in Vietnam, and whether poultry-associated viruses in Laos may represent an undercharacterized reservoir relevant to future spillover risk (8, 11, 12).

A critical gap in existing surveillance is the relationship between sequence variation and functional adaptation. In HPAI H5 viruses, substitutions in the PB2 gene and the hemagglutinin (HA) head domain are important determinants of mammalian adaptation and interspecies transmission (1, 2, 13). Experimental studies have shown that avian H5N1 viruses carrying PB2-627E can acquire the mammalian-adaptive PB2-E627K substitution during replication in mammalian hosts, and that this change is associated with enhanced viral replication (9, 10, 14). In addition, specific HA receptor-binding-site substitutions can increase recognition of α2,6-linked sialic acid receptors, which are characteristic of the human upper respiratory tract (15, 16). Integrating genomic data with structural modeling and receptor-binding analysis is therefore important for evaluating the zoonotic potential of avian H5 strains before they acquire stable mammalian-adaptive traits (17).

In this study, we report the genomic characterization of eight H5N1 clade 2.3.2.1e viruses isolated from live bird markets (LBMs) in Lao PDR during 2023–2024. Using next-generation sequencing and in silico structural modeling, we investigated their phylogenetic placement relative to recent regional human infections and tiger/leopard mortality events reported in Cambodia and Vietnam (8, 11). We also analyzed molecular signatures associated with mammalian adaptation, including PB2 and HA markers (9, 10, 14). Through homology modeling and Molecular Mechanics-Generalized Born Surface Area (MM-GBSA) calculations, we further evaluated the predicted receptor-binding properties of a representative Lao isolate.

Accordingly, this study aimed to characterize the complete genomes and phylogenetic relationships of the Lao H5N1 isolates, identify molecular markers associated with mammalian adaptation and pathogenicity, and evaluate the predicted binding affinity of a representative isolate for avian-type α2,3-linked and human-type α2,6-linked sialic acid receptors. These findings are expected to provide genomic and structural evidence that may contribute to One Health surveillance and pandemic preparedness in the Greater Mekong Subregion.

## 2 Materials and methods

### 2.1 Sample collection and source of specimens

Active surveillance was conducted between 2023 and 2024 in live bird markets across five provinces of Lao PDR: Bokeo, Luangnamtha, Oudomxay, Vientiane Capital, and Salavan. A total of 32 poultry-derived and environmental samples, including specimens collected from ducks, chickens, geese, and market environments, were obtained through routine field surveillance activities. The number of samples collected in each province was as follows: Bokeo (*n* = 4), Luangnamtha (*n* = 3), Oudomxay (*n* = 10), Vientiane Capital (*n* = 14), and Salavan (*n* = 1). The specimens were officially provided by the National Animal Health Laboratory (NAHL), Department of Livestock and Fisheries, Ministry of Agriculture and Forestry, Lao PDR. Detailed metadata for all samples, including the collection date, geographic origin, host species or source, sample type, are summarized in Supplementary Table S1.

### 2.2 Virus isolation and identification

For virus isolation, processed samples were inoculated into the allantoic cavity of 10-day-old specific-pathogen-free (SPF) embryonated chicken eggs. Inoculated eggs were incubated at 37 °C for 72–96 h. After incubation, allantoic fluids were harvested and screened for hemagglutinating activity using chicken red blood cells. Eight HA-positive isolates were recovered from 32 samples, including five from Vientiane Capital and one each from Bokeo, Luangnamtha, and Oudomxay. All virus isolation and subsequent experiments involving infectious avian influenza viruses were conducted in the certified biosafety level 3 facility at the Korea Zoonosis Research Institute (KoZRI), Jeonbuk National University, Republic of Korea, under approval from the Jeonbuk National University Institutional Biosafety Committee (JBNU 2024-09-003-001, 2025-07-001).

**Table 1:** Eight H5N1 influenza viruses showing highest nucleotide identity across the whole genome.

Segment	Closest virus	Nucleotide identity	Accession No.
PB2	A/chicken/Ehime/TU11-2-24,25/2022 (H5N1)	98.8–99.06%	LC699661.1
PB1	A/Bean Goose (*Anser fabalis*)/South Korea/KNU2021-42/2021(H1N1)	97.7–98.5%	ON495895.1
PA	A/duck/Ilan/19WB0251-20-E1-2D/2019(H12N1)	97.2–97.9%	OR949355.1
HA	A/Bird/Indonesia/EPI_ISL_HA19613397/2022(H5N1)	98.7–99.6%	PV422429.1
NP	A/duck/Vietnam/HU10-1757/2018(H5N1)	97.6–98.2%	LC500423.1
NA	A/duck/Vietnam/HU10-1757/2018(H5N1)	97.4–99.7%	LC500424.1
M	A/chicken/Viet Nam/LBFecal221LC/2022(H5N1)	99.1–99.9%	PV410632.1
NS	A/chicken/Hokkaido/TU25-3/2022(H5N1)	98.3–99.3%	LC718366.1

HA, haemagglutinin; M, matrix protein; NA, neuraminidase; NP, Nucleoprotein; NS, non-structural protein; PB1, polymerase basic 1; PB2, polymerase basic 2; PA, polymerase acidic.

### 2.3 RNA extraction and whole-genome sequencing

Total RNA was extracted from hemagglutination-positive allantoic fluids using either the Patho Gene-spin Viral DNA/RNA Extraction Kit or the EZ1 Virus Mini Kit, as previously described (18, 19) with minor modifications. Whole-genome sequencing was performed on an Illumina platform to generate consensus sequences for all eight influenza gene segments.

### 2.4 Sequence dataset construction and phylogenetic analysis

Reference HA and whole-genome sequences representing clades 2.3.2.1e, 2.3.2.1c, 2.3.4.4b, and 2.3.4.4 h were retrieved from the GISAID EpiFlu database and NCBI GenBank. Sequence alignment was performed using MAFFT version 7 (20). Maximum-likelihood phylogenetic trees were constructed using MEGA version 12.0 (21) to further investigate temporal relationships among selected sequences, time-scaled phylogenetic analysis was conducted in BEAST v1.10.4 (22) using the HKY + G nucleotide substitution model (23), an uncorrelated lognormal relaxed molecular clock (24), and a coalescent Gaussian Markov Random Field (GMRF) Bayesian Skyride tree prior (25).

### 2.5 Comparative genetic analysis and molecular marker profiling

The Lao isolates were compared with representative H5N1 viruses associated with recent human infections in Cambodia and Vietnam and with tiger-associated H5N1 viruses reported from Vietnam. Molecular analysis focused on known markers associated with host adaptation and pathogenicity, including PB2 residues 627 and 701, the HA receptor-binding region, and the polybasic HA cleavage-site motif. Multiple alignments of the deduced amino acid sequences were generated using Clustal Omega version 1.2.4 (26), and conserved and variable amino acid residues were visualized using ESPript 3.0 (27). The analyses were conducted using the default parameters of each program.

**Table 2:** Comparison of HA cleavage-site motifs and selected functional HA1 marker residues among H5 sequences.

Contents	Accession/ID	Cleavage site	HA1-94 D94N	HA1-123 S123P	HA1-154 N154D	HA1-188 T188I	HA1-223 Q-S223	HA1-263 A263T	HA1-497 R497K
V Tiger	EPI_ISL_20081455	PQRERRRKRGLF	N	S	D	T	S	T	L
	EPI_ISL_19520689	PQRERRRKRGLF	N	S	D	T	S	T	L
	EPI_ISL_19520627	PQRERRRKRGLF	N	S	D	T	S	T	L
	EPI_ISL_19520622	PQRERRRKRGLF	N	S	D	T	S	T	L
	EPI_ISL_19520621	PQRERRRKRGLF	N	S	D	T	S	T	L
V human	EPI_ISL_19031556	PQREGRRKRGLF	N	S	N	T	S	T	L
	EPI_ISL_19850663	PQREGRRKRGLF	N	P	N	T	S	T	K
C human	EPI_ISL_19312044	PQRERRRKRGLF	N	S	N	T	S	T	L
	EPI_ISL_18823967	PQRERRRKRGLF	N	S	D	T	S	T	L
	EPI_ISL_18939130	PQREGRRKRGLF	N	S	N	T	S	T	R
	EPI_ISL_18543642	PQRERRRKRGLF	N	S	D	T	S	T	L
L avian	EPI_ISL_19678907	PQRERRRKRGLF	N	S	N	M	S	T	L
	EPI_ISL_19,678,908	PQRERRRKRGLF	N	S	N	M	S	T	L
	EPI_ISL_19678909	PQRERRRKRGLF	N	S	N	M	S	T	L
	EPI_ISL_20050893	PQRERRRKRGLF	N	S	N	T	G	T	L
	EPI_ISL_20459161	PQRERRRKRGLF	N	S	N	T	G	T	L
	EPI_ISL_20459162	PQRERRRKRGLF	S	S	N	T	S	T	L
	EPI_ISL_20459163	PQRERRRKRGLF	S	S	N	T	S	T	L
	EPI_ISL_20459156	PQRERRRKRGLF	S	S	N	T	S	T	L

Representative H5 HA sequences from Vietnamese tiger isolates, Vietnamese human cases, Cambodian human cases, Lao avian isolates, and a low-pathogenic avian influenza reference sequence were compared at selected HA1 amino acid positions.

Geographic locations of H5N1 detections in Lao PDR and selected human and mammalian H5N1 events in neighboring countries were plotted to visualize the regional distribution of clade 2.3.2.1e. The map included northern Lao provinces, including Luangnamtha, Bokeo, and Oudomxay, Vientiane Capital, and selected mammalian event sites in Cambodia and Vietnam. Geographic maps showing the sampling locations were generated using QGIS version 3.44.7 (28).

### 2.6 Homology modeling of the HA protein

A representative Lao avian isolate, EPI_ISL_19678908, was selected for structural analysis of the HA protein. The full-length HA amino acid sequence, consisting of 565 amino acids, was modeled using the SWISS-MODEL automated homology modeling server (https://swissmodel.expasy.org, accessed 20 March 2026). The crystal structure of A/Hubei/1/2010 (H5N1), PDB ID: 4KTH, which belongs to clade 2.3.2.1, was used as the modeling template because of its high sequence similarity to the Lao isolate. Model quality was evaluated using the global assessment metrics provided by SWISS-MODEL. For the selected model, Chain A, which contains the receptor-binding site (RBS), exhibited a GMQE score of 0.44 and a QMEANDisCo Global score of 0.77 ± 0.05, while Chain B yielded a GMQE of 0.18 and a QMEANDisCo Global score of 0.66 ± 0.07. Structural similarity between the modeled Lao HA and the template structure was further evaluated using root mean square deviation (RMSD).

### 2.7 Receptor preparation and MM-GBSA binding free energy analysis

To assess host receptor-binding characteristics, avian-type and human-type sialic acid receptor analogs were analyzed using computational approaches. Sialic acid ligand coordinates were obtained from H1 influenza hemagglutinin crystal structures deposited in the RCSB Protein Data Bank. The α2,3-linked sialic acid analog, 3′-SLN, was obtained from PDB ID 3UBQ, and the α2,6-linked sialic acid analog, 6′-SLN, was obtained from PDB ID 3UBN. Proteins were processed using the Protein Preparation Wizard in Schrödinger Suite 2025–1 with default options. To avoid artificial structural biases often introduced by molecular docking scoring functions, receptor-ligand complexes were constructed by structural overlay; prepared HA structures (3UBQ, 3UBN, and 4KTH) were structurally aligned based on C-alpha atoms, and the ligand coordinates of 3′-SLN and 6′-SLN were extracted and transferred onto the 4KTH receptor-binding site. The same ligand placement strategy was applied to the EPI_ISL_19678908 HA homology model after structural alignment with the 4KTH template.

Binding free energies (Δ*G*_bind_) were calculated using Prime MM-GBSA in Schrödinger Suite 2025–1, employing the VSGB solvation model and the OPLS4 force field. To accommodate local induced-fit conformational changes and resolve potential steric clashes resulting from structural alignment, residues located within 6.0 Å of the ligand were treated as flexible during the minimization and energy calculation steps, with the sampling method set to minimization.

### 2.8 Hydrogen bond interaction analysis

Hydrogen bond interactions between the HA receptor-binding site and the receptor analogs were analyzed and visualized using Maestro in Schrödinger Suite 2025–1. The number of hydrogen bonds, the distribution of interacting residues, and hydrogen bond distances were evaluated to compare the receptor interaction patterns of the Lao isolate with those of the reference human-infecting H5 structure, A/Hubei/1/2010.

### 2.9 Predicted immunogenicity

The HA amino acid sequences of A/Hubei/1/2010 (EPI_ISL_96896) and A/duck/Vientiane/C2431/2023 (EPI_ISL_19678908) were subjected to comparative in silico prediction of MHC class I- and class II-restricted T-cell epitopes using complementary tools available through the Immune Epitope Database and Analysis Resource.

For MHC class I analysis, peptide-binding candidates were initially screened using the IEDB MHC I Binding Prediction tool version 2023.09 against the full HLA reference set, with peptide lengths of 9–10 amino acids and a percentile-rank threshold of ≤0.1. Antigen processing and presentation were subsequently evaluated using the IEDB MHC I Processing Prediction tool with the IEDB-recommended method for HLA-A, HLA-B, and HLA-C alleles. Nine-mer peptides with a total processing score of ≥1 were retained. The likelihood of natural MHC class I presentation was further assessed using MHC-NP for HLA-A02:01, HLA-B07:02, HLA-B35:01, HLA-B44:03, HLA-B53:01, and HLA-B57:01, considering 9–11-mer peptides with a probability score of ≥0.8.

For MHC class II analysis, binding peptides were predicted using the IEDB MHC II Binding Prediction tool with the IEDB-recommended 2.22 method against the full HLA reference set. Twelve-mer peptides with a percentile rank of ≤0.15 were selected. The predicted candidates were additionally evaluated using the MHC-II NP prediction and CD4 T-cell immunogenicity prediction tools. Predicted epitope-associated regions were mapped to the corresponding HA amino acid positions and compared between the two viruses.

## 3 Results

### 3.1 Sample collection and virus isolation

Between 2023 and 2024, active surveillance was conducted in live bird markets across five provinces in Lao PDR, namely Bokeo, Luangnamtha, Oudomxay, Vientiane Capital, and Salavan. A total of 32 environmental and poultry-derived samples, including samples from ducks and geese, were collected and processed for virus detection and isolation. Following propagation in specific-pathogen-free embryonated chicken eggs and confirmation by hemagglutination assay, eight H5N1 highly pathogenic avian influenza viruses were successfully isolated. The recovered isolates originated from Vientiane Capital (*n* = 5), Bokeo (*n* = 1), Luangnamtha (*n* = 1), and Oudomxay (*n* = 1).

### 3.2 Phylogenetic placement of Lao isolates within clade 2.3.2.1e

**Figure 1:**
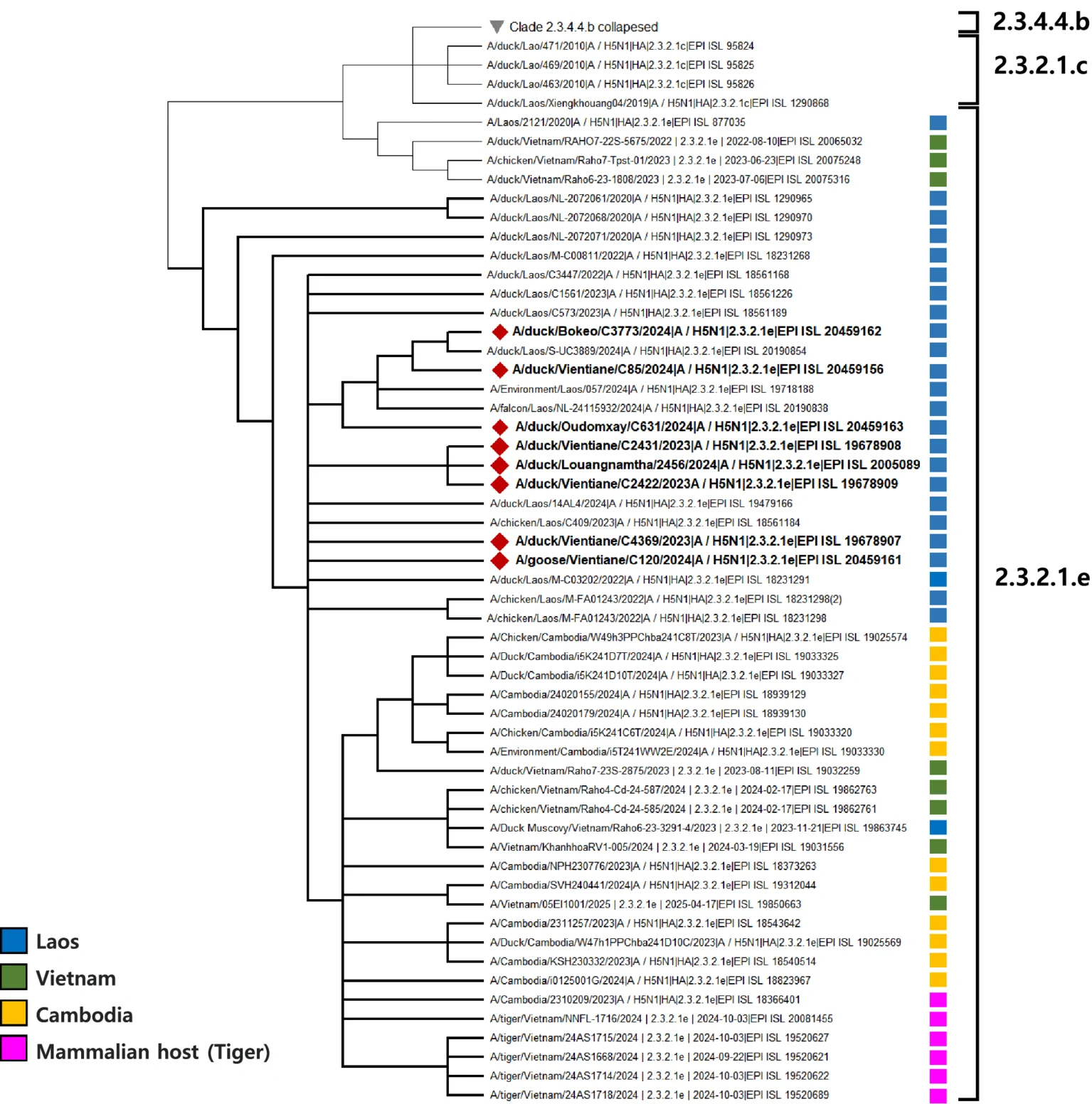
Maximum-likelihood phylogenetic tree of the hemagglutinin (HA) gene of H5N1 highly pathogenic avian influenza viruses (HPAI) circulating in Southeast Asia (2020–2025). The tree was constructed using HA gene sequences of H5N1 viruses retrieved from the GISAID EpiFlu database, including isolates obtained in this study and representative reference strains. Reference clades 2.3.4.4b (collapsed) and 2.3.2.1c are shown at the top of the tree, while the lower portion comprises isolates belonging to clade 2.3.2.1e, which represents the predominant lineage currently circulating in Laos, Vietnam, and Cambodia. Red diamonds (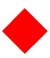) indicate the eight novel H5N1 isolates characterized in this study, collected from domestic ducks and geese across multiple provinces of Laos (Vientiane, Bokeo, Oudomxay, and Louangnamtha) during 2023–2024. Colored squares on the right side of the tree denote the geographic origin and host species of each isolate.

To characterize the genetic relationships of H5N1 highly pathogenic avian influenza (HPAI) viruses circulating in Laos, we performed a maximum-likelihood phylogenetic analysis based on the HA gene sequences of eight isolates obtained from domestic poultry between 2023 and 2024, together with regional reference strains retrieved from the GISAID EpiFlu database (Figure 1, Supplementary Figure S6).

All eight Laotian isolates characterized in this study (indicated by red diamonds, 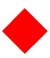) were classified into clade 2.3.2.1e, forming a well-supported monophyletic group distinct from the reference clades 2.3.4.4b and 2.3.2.1c. The clade 2.3.2.1c reference branch contained historical Laotian isolates from 2010 (A/duck/Lao/463, 469, 471/2010) along with a limited number of Vietnamese and Laotian sequences collected between 2019 and 2023, indicating that a clade replacement event from 2.3.2.1c to 2.3.2.1e has occurred in the Laotian poultry population in recent years.

The novel isolates were collected from geographically diverse provinces of Laos, including Vientiane (*n* = 5), Bokeo (*n* = 1), Oudomxay (*n* = 1), and Louangnamtha (*n* = 1), and were obtained from ducks (*n* = 7) and one goose. Within clade 2.3.2.1e, these isolates resolved into two distinct sub-clusters:

Sub-cluster I comprised A/duck/Bokeo/C3773/2024 and A/duck/Vientiane/C85/2024, which clustered closely with an environmental isolate (A/Environment/Laos/057/2024) and a falcon-derived isolate (A/falcon/Laos/NL-24115932/2024), suggesting interfaces among poultry, environment, and wild bird ecosystems.

**Figure 2:**
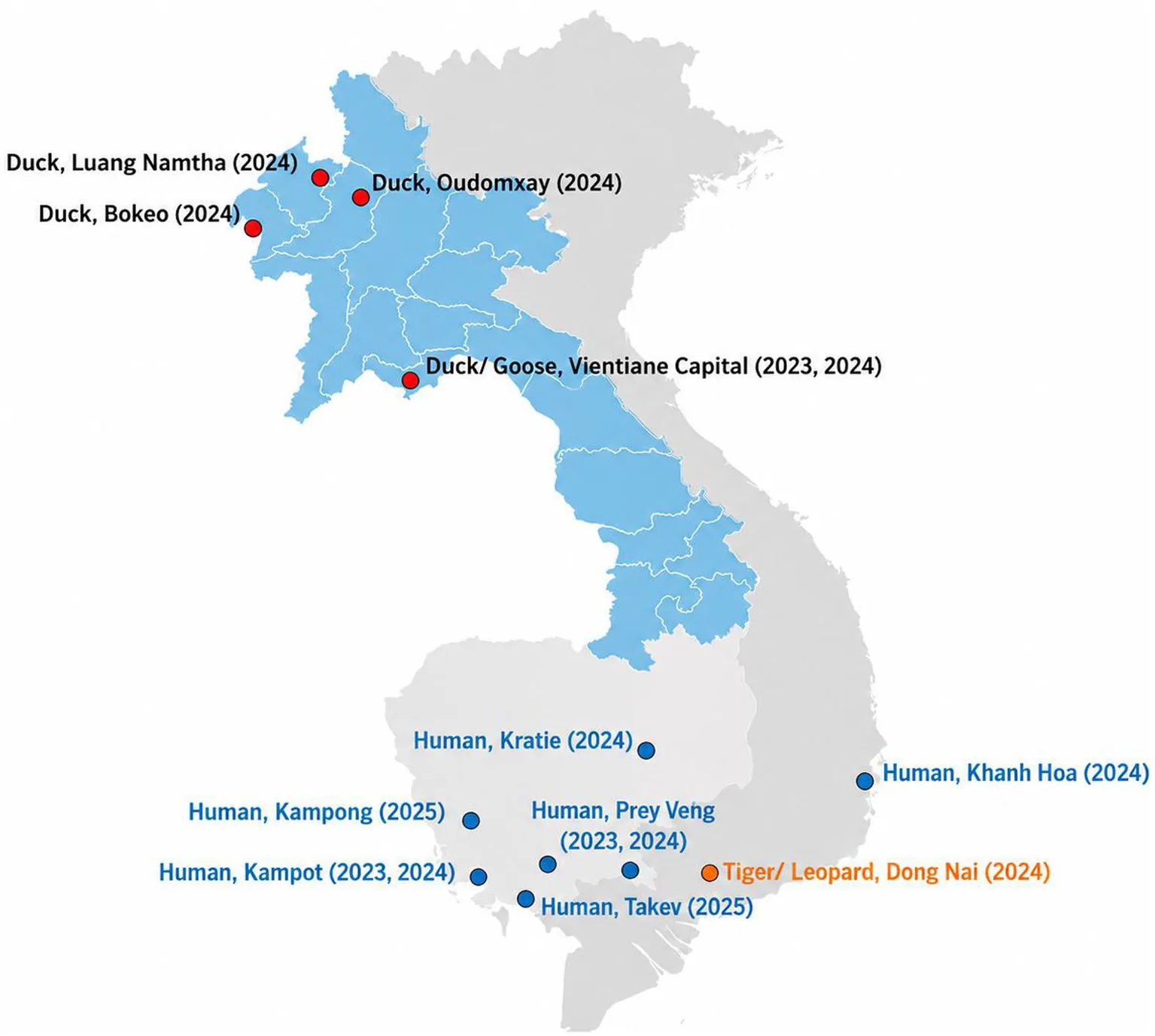
Geographic distribution of Lao PDR LBM-derived H5N1 isolates (2023–2024) and locations of recent regional mammalian cases. Avian isolates were detected in northern (Luang Namtha, Bokeo, Oudomxay) and central (Vientiane Capital) Lao PDR. Human cases in Cambodia and Vietnam, and the tiger/leopard outbreak in Dong Nai, Vietnam, are shown for regional context.

Sub-cluster II included A/duck/Oudomxay/C631/2024, A/duck/Vientiane/C2431/2023, A/duck/Louangnamtha/2456/2024, and A/duck/Vientiane/C2422/2023, which exhibited very short branch lengths, indicating a recent common ancestor and rapid intra-country dissemination across northern and central Laos.

Notably, the Laotian isolates were intermingled with contemporaneous H5N1 viruses from Vietnam (teal) and Cambodia (orange) within clade 2.3.2.1e, and shared close phylogenetic proximity with mammalian-host isolates obtained from tigers in Vietnam (A/tiger/Vietnam/24AS1715, 1,668, 1714/2024; shown in purple). These findings strongly suggest transboundary circulation of clade 2.3.2.1e viruses across the Mekong region and highlight the spillover potential of this lineage from avian to mammalian hosts.

### 3.3 Geographic distribution of Lao avian detections and relationship to regional mammalian events

Spatial mapping showed that the Lao H5N1 detections were distributed across both northern and central parts of the country. Specifically, positive isolates were identified in Luangnamtha, Bokeo, and Oudomxay in northern Lao PDR and in Vientiane Capital in the central region. When examined alongside recent H5N1-associated mammalian and human events in neighboring countries, these detections aligned geographically with a wider regional pattern of clade 2.3.2.1e circulation. The Lao market-derived viruses were therefore considered in the context of recent fatal human infections reported in Cambodia and Vietnam and the notable tiger and leopard mortality event reported in Dong Nai, Vietnam (Figure 2).

### 3.4 Whole-genome identity and genotype constellation

Whole-genome sequence comparison demonstrated that the Lao isolates shared high nucleotide identity with previously reported avian influenza viruses, with segment-level similarities ranging from 97.2 to 99.9%. No single reference virus represented the closest match across all eight genomic segments, indicating that the Lao viruses possessed a complex genotype constellation rather than a uniform genomic background. The PB2 segment showed the highest identity to a 2022 H5N1 virus from Ehime, Japan (98.8–99.06%), whereas the HA segment was most closely related to a 2022 H5N1 virus reported from Indonesia (98.7–99.6%) (Table 1). Other internal and surface segments were most similar to viruses reported from South Korea, Vietnam, Iran, and Japan, including PB1-related similarity to a bean goose-origin H1N1 virus from South Korea and PA-related similarity to an H12N1 duck virus from Iran. Together, these findings indicate that the Lao isolates were associated with a mosaic regional genetic background.

### 3.5 Non-HA segment marker analysis reveals avian-like internal gene profiles and Lao-associated variation in H5N1 clade 2.3.2.1e viruses

Amino-acid sequences of seven non-HA segments were compared between Lao H5N1 clade 2.3.2.1e isolates and reference sequences from Vietnamese tiger, Vietnamese human, and Cambodian human cases. Positions were summarized as known functional markers, group-associated substitutions, or Lao-associated/within-Lao variable sites.

For PB2, all analyzed proteins were 759 aa in length, with 48 variable positions. Lao isolates showed PB2-389R, mixed PB2-508 residues, PB2-627E, and mixed PB2-655 V/L patterns. Major mammalian-adaptive PB2 markers, including T271A, K526R, A588V, G590S/Q591K/R, E627K, M631L, D701N, and S714R/I, were not detected in Lao isolates. All Lao isolates retained PB2-627E and PB2-701D, whereas many reference sequences carried PB2-627 K (Supplementary Table S2, Figure S3).

For PB1, all proteins were 757 aa, with 30 variable positions. Major PB1 polymerase-associated markers, including N105S, K577E, and S678N, were not detected in Lao isolates. PB1-D622G was conserved in both reference and Lao sequences. Lao isolates consistently carried PB1-204T and PB1-222 L, whereas reference sequences retained PB1-204I and PB1-222M. Additional within-Lao variation was observed at PB1-54, PB1-110, PB1-187, and PB1-337 (Supplementary Table S3, Figure S4).

For polymerase acidic protein (PA), all analyzed proteins were 716 aa, with 37 variable positions. Major PA mammalian-adaptive substitutions, including T97I, S224P, A343S, D347E, and S409N, were not detected in Lao isolates. PA-N383D was conserved in both reference and Lao sequences. Lao PA sequences shared PA-142 K, PA-337A, and PA-418 T, while reference sequences predominantly retained PA-142 N, PA-337 T, and PA-418I. Within-Lao variation was observed at PA-210 and PA-409. One Lao isolate, EPI_ISL_19678909, was marked as ND in the PA table because its PA sequence was not available in the provided PA dataset (Supplementary Table S4, Figure S5).

For nucleoprotein (NP), all proteins were 498 aa, with 13 variable positions. NP-105 V and NP-184 K were conserved in both reference and Lao sequences. Major NP mammalian-adaptation or virulence-associated substitutions, including N319K, T437M, and K470R, were not detected in Lao isolates. Vietnamese tiger sequences showed NP-119M, NP-432G, and NP-446 K, whereas Lao isolates retained NP-119I, NP-432S, and NP-446R. Within-Lao variation was observed at NP-129 and NP-350 (Supplementary Table S5, Figure S7).

For neuraminidase (NA), all proteins were 449 aa, with 19 variable positions. Major neuraminidase inhibitor-resistance-associated substitutions, including E119V, Q136K, D151G/A, R152K, I223V/T/R/K, H275Y, and N295S, were not detected in Lao isolates. Vietnamese tiger sequences showed group-associated patterns such as NA-6R, NA-220 T, and NA-353I, whereas Lao isolates retained NA-6K, NA-220A, and NA-353 M. Lao-associated variation was most evident at NA-129A/I and NA-293H/Q (Supplementary Table S6, Figure S8).

For matrix protein (MP), M segment (M)1 and M2 were analyzed separately. M1 was 252 aa and M2 was 97 aa. The reference dataset contained M1 sequences only, whereas the Lao dataset contained both M1 and M2 sequences. Therefore, M1 was compared between reference and Lao isolates, while M2 was evaluated only among Lao isolates. In M1, known residues M1-30D, M1-43M, and M1-215A were conserved. M1 variation was limited to M1-115 and M1-224. For M2, known adamantane-resistance markers, including V27A, A30T/V, S31N, and G34E, were not detected, but within-Lao variation was observed at M2-12, M2-28, M2-42, M2-70, and M2-82 (Supplementary Table S7, Figure S9).

For non-structural protein (NS), only NS1 was analyzed because NS2/NEP sequences were not included in the provided datasets. Lao NS1 proteins were 230 aa, and 34 variable positions were identified. One Vietnamese human reference sequence, EPI_ISL_19031556, showed an NS1-Δ80–84 deletion, but this deletion was not observed in Lao isolates. NS1-42S, NS1-92D, NS1-106M, NS1-149A, and the C-terminal ESEV PDZ-binding motif were conserved in Lao isolates. Lao sequences retained NS1-138F, shared NS1-207N, and showed internal variation at NS1-103 and NS1-215 (Supplementary Table S8, Figure S10).

ND indicates that the residue could not be determined because the corresponding amino-acid sequence was not available in the provided dataset. M2 reference residues and NS2/NEP residues were therefore not interpreted as absent mutations, but as unavailable data.

### 3.6 HA-centered marker analysis reveals receptor-binding-related molecular signatures in Lao H5N1 clade 2.3.2.1e viruses

Among the eight Lao avian H5 isolates, all sequences shared the same polybasic HA cleavage motif, PQRERRRKRGLF. They also showed identical residues at several functional HA1 marker positions, including HA1-123S, HA1-154N, HA1-263T, and HA1-497L. However, sequence variation was observed at HA1-94, HA1-188, and HA1-223, allowing the Lao avian isolates to be divided into three marker-pattern groups (Table 2). The first group included EPI_ISL_19678907, EPI_ISL_19678908, and EPI_ISL_19678909. These three isolates shared an identical marker pattern, characterized by HA1-94N, HA1-188M, and HA1-223S. This group was distinct from the other Lao avian isolates by the presence of M at HA1-188. The second group included EPI_ISL_20050893 and EPI_ISL_20459161. These isolates showed HA1-94 N, HA1-188T, and HA1-223G. Compared with the first group, this group retained T instead of M at HA1-188 and showed G instead of S at HA1-223.

The third group included EPI_ISL_20459162, EPI_ISL_20459163, and EPI_ISL_20459156. These isolates shared HA1-94S, HA1-188T, and HA1-223S. This group differed from the first and second groups mainly by the presence of S at HA1-94, while retaining T at HA1-188 and S at HA1-223.

The eight Lao avian isolates showed a conserved highly pathogenic HA cleavage motif and shared common residues at HA1-123, HA1-154, HA1-263, and HA1-497. Nevertheless, they were not completely identical and could be separated into three HA marker-pattern groups based on variation at HA1-94, HA1-188, and HA1-223.

### 3.7 Structural modeling of the receptor-binding region

**Figure 3:**
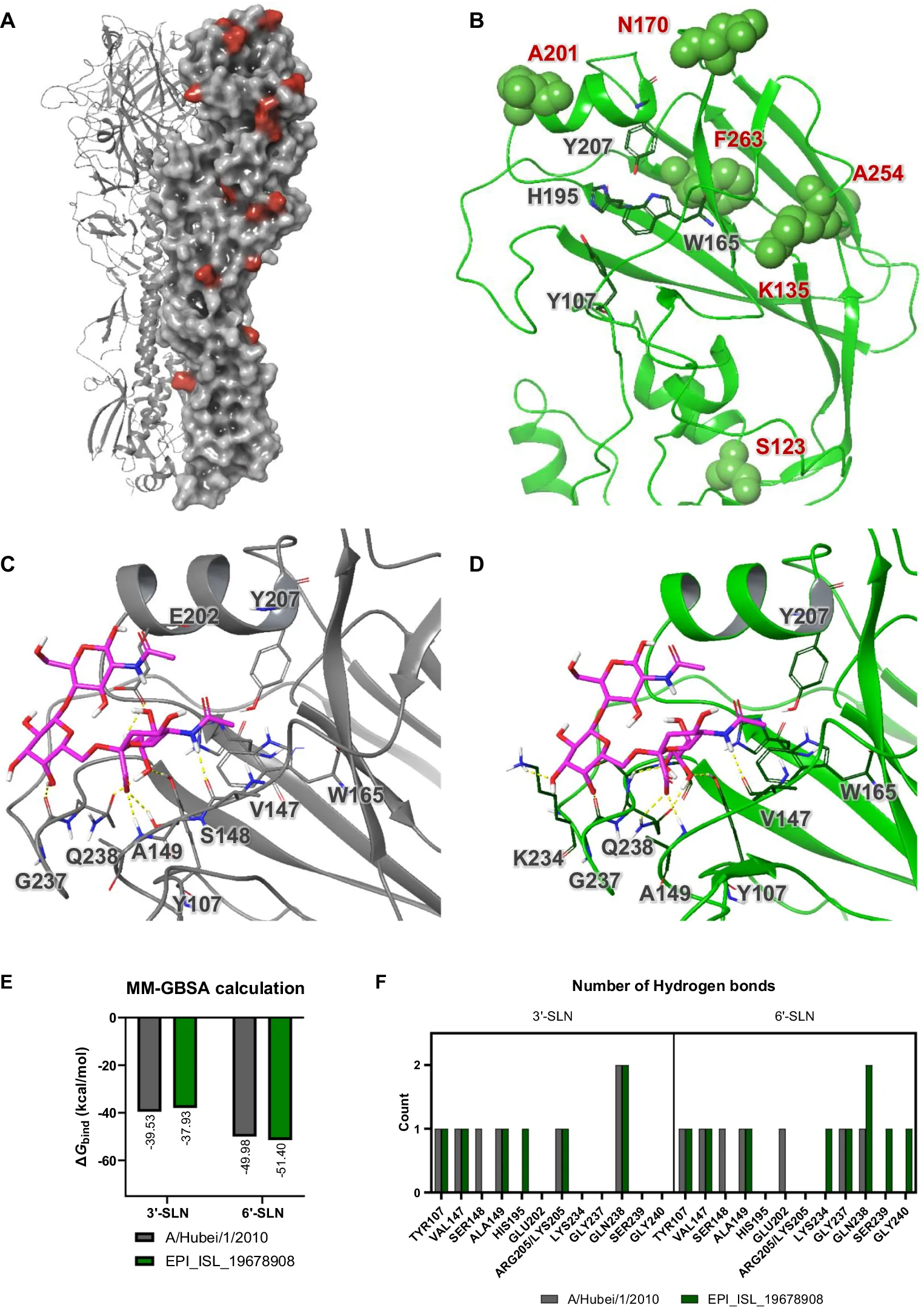
Structural modeling and comparative analysis of Lao PDR H5 HA with human-infectible H5N1. Molecular mapping of amino acid variation. **(A)** The functional trimer of the human-isolated reference strain, A/Hubei/1/2010 (PDB ID: 4KTH), is displayed as gray ribbons. One monomer is rendered as a gray surface, with the residue positions that differ in the Lao PDR avian isolates highlighted in red. Close-up view of the globular head. **(B)** The structural features of the EPI_ISL_19678908 HA are depicted in a green ribbon. Conserved sialic acid-binding pocket residues are shown as dark green sticks with gray labels. The key functional HA marker residues are presented as green spheres with red labels. **(C,D)** Molecular interactions in the receptor-binding site with 6’-SLN. Close-up view of the receptor-binding pocket of A/Hubei/1/2010 (gray ribbon, **C**) and EPI_ISL_19678908 (green ribbon, **D**) in complex with 6’-SLN (magenta sticks). Key residues are shown as sticks and labeled. Yellow dashed lines indicate hydrogen bond interactions. **(E)** MM-GBSA binding free energies (Δ*G*_bind_, kcal/mol) of A/Hubei/1/2010 (gray) and EPI_ISL-19678908 (green) with 3’-SLN and 6’-SLN ligands. **(F)** Number of hydrogen bonds formed between each receptor-binding site residue and the sialic acid analog 3’-SLN (left) and 6’-SLN (right) for A/Hubei/1/2010 (gray) and EPI_ISL-19678908 (green).

To explore the structural implications of the observed HA variation, a representative Lao isolate (EPI_ISL_19678908) was selected for homology modeling using the human-infecting H5 reference strain A/Hubei/1/2010 (PDB ID: 4KTH) as the template. The resulting homology model exhibited high sequence identity (96%) to the template and demonstrated high structural quality, with a QMEANDisCo Global score of 0.77 ± 0.05 and a GMQE of 0.44 for Chain A, which contains the receptor-binding site (RBS). The modeled Lao HA structure showed a minimal root mean square deviation (RMSD) of 0.2794Å relative to the template, indicating a overall close backbone match (Supplementary Figure S1). Mapping of the identified HA markers onto the three-dimensional model showed that several of the variable residues were located within or adjacent to the receptor-binding region, including positions with potential relevance to local topology, electrostatic environment, and steric accessibility of the receptor-binding pocket (Figures 3A,B).

### 3.8 MM-GBSA binding free energy and hydrogen bond analysis

To quantitatively evaluate host receptor-binding affinities, Prime MM-GBSA calculations were performed on the structural overlay-constructed receptor complexes involving avian-type (3′-SLN) and human-type (6′-SLN) receptor analogs (Figures 3C,D; Supplementary Figures S2A,B). For the representative Lao isolate, the predicted binding free energy (Δ*G*_bind_) was −37.93 kcal/mol for the avian-type α2,3-linked receptor analog (3′-SLN) and −51.40 kcal/mol for the human-type α2,6-linked receptor analog (6′-SLN) (Figure 3E). For the A/Hubei/1/2010 reference, the corresponding values were −39.53 kcal/mol and −49.98 kcal/mol, respectively. Thus, the Lao avian isolate showed a stronger predicted interaction with the human-type receptor analog than with the avian-type analog and exhibited a human-type receptor-binding energy profile that approached that of the zoonotic reference strain. To validate the specificity of this thermodynamic trend within our modeling framework, parallel MM-GBSA calculations were conducted on a low-pathogenic avian reference strain, A/Duck/Singapore/3/1997 (PDB ID: 1JSM) (Supplementary Figure S2C). In sharp contrast, the low-pathogenic avian control demonstrated the expected classical avian preference, showing stronger predicted binding to 3′-SLN (−34.43 kcal/mol) than to 6′-SLN (−31.57 kcal/mol). Hydrogen bond analysis further showed that the Lao isolate formed a greater total number of hydrogen bond interactions with the α2,6-linked receptor analog, although the spatial distribution and specific participating residues differed from that of the reference structure (Figures 3C,D,F; Supplementary Table S9).

### 3.9 Comparative analysis of predicted T-cell immunogenicity in H5N1 HA proteins

Comparative analysis identified three MHC class I-associated epitope regions in the HA protein of A/Hubei/1/2010 and four regions in A/duck/Vientiane/C2431/2023 Two predicted MHC class I-associated regions were conserved between the viruses, including IAPEY at positions 264–268 and MPFHNIHPL at positions 305–313. In contrast, the region spanning positions 205–208 differed between the two HA sequences, with RLYQ identified in A/Hubei/1/2010 and KLYQ identified in A/duck/Vientiane/C2431/2023. This difference corresponded to an R205K amino acid substitution within the predicted epitope-associated region. An additional region, KSDHIC at positions 15–20, was identified exclusively in the Lao isolate For MHC class II, the same predicted epitope sequence, EWSYIVEKANPAN, was identified in both viruses, indicating conservation of this MHC class II-associated region. The predicted T-cell epitope profiles were largely conserved between the two HA proteins, although isolate-specific variation was observed within the MHC class I-associated regions (Table 3; Supplementary Figures S11, S12).

**Table 3:** Comparison of predicted MHC-binding peptides in H5N1 HA proteins.

MHC class	Position	A/Hubei/1/2010 (EPI_ISL_96896)	A/duck/Vientiane/C2431/2023 (EPI_ISL_19,678,908)
MHC-I	15–20	—	KSDHIC
	205–208	RLYQ	KLYQ
	264–268	IAPEY	IAPEY
	305–313	MPFHNIHPL	MPFHNIHPL
MHC-II	91–102	EWSYIVEKANPAN	EWSYIVEKANPAN

## 4 Discussion

The present genomic findings provide evidence for the ongoing circulation of H5N1 clade 2.3.2.1e viruses in poultry-associated live bird markets in Lao PDR, underscoring the need for sustained market-based surveillance in the region. This finding is consistent with recent surveillance data from Laos, which reported avian influenza virus positivity rates of 72% in live ducks and 18% in live chickens, together with the detection of HPAI H5N1 clade 2.3.2.1e viruses in market-associated samples (5). These high positivity rates underscore the active circulation of avian influenza viruses in Lao live animal markets. Building on previous prevalence-based surveillance, the present study provides whole-genome characterization of eight H5N1 clade 2.3.2.1e viruses collected from multiple Lao provinces during 2023–2024 and places them within the broader regional evolutionary context of the Indochinese Peninsula.

The regional relevance of clade 2.3.2.1e is further underscored by recent human cases and mammalian outbreaks of H5N1 in neighboring countries. Since 2023, human H5N1 infections have re-emerged in Cambodia, with reported case-fatality estimates ranging from approximately 38 to 44.4%, depending on the reporting period (10, 15). Recent assessments and outbreak reports have also linked clade 2.3.2.1e viruses to human infections in Cambodia and mammalian outbreaks in Vietnam, including captive wild felids (11, 29). Amano et al. (8) reported that tiger and leopard associated Vietnamese H5N1 viruses belonged to clade 2.3.2.1e and carried mammalian-adaptation-associated mutations, including PB2-E627K. Consistent with these reports, our Lao isolates were positioned within clade 2.3.2.1e and were related to recent regional viruses. Notably, the Lao isolates did not form a single country-specific monophyletic lineage but were distributed among multiple phylogenetic sublineages, indicating that they are part of a broader regional transmission and evolutionary network rather than a discrete, locally confined market-associated cluster.

The present study builds on previous surveillance by revealing the complex segment-level genomic architecture of Lao H5N1 viruses. Although earlier market-based studies documented AIV circulation and detected clade 2.3.2.1e viruses in Laos, the whole-genome relationships among contemporary Lao isolates remained poorly characterized (5). Segment-wise sequence analysis identified different closest relatives across the eight gene segments, revealing a mosaic genomic constellation consistent with regional gene flow and historical reassortment among viruses from diverse geographic regions and avian hosts.

PB2 residue 627 is a key determinant of H5N1 virulence in mammalian hosts, and avian H5N1 viruses carrying PB2-627E can rapidly acquire PB2-E627K during replication in mice (10, 14). Amano et al. (8) also reported that tiger and leopard associated H5N1 viruses from Vietnam carried PB2-E627K and other mammalian-adaptation-associated mutations. In contrast, all eight Lao poultry-derived isolates in this study retained the avian-associated PB2-627E and PB2-701D residues, indicating that they had not acquired the classical mammalian-adaptive substitutions PB2-E627K or PB2-D701N. However, the absence of these canonical PB2 mutations should not be interpreted as evidence that the Lao isolates pose no zoonotic risk. PB2-E627K may emerge rapidly following spillover and replication in mammalian hosts, and mammalian adaptation is a multigenic process that can also be influenced by changes in HA receptor binding, other polymerase proteins, and additional viral gene segments. Therefore, the current PB2 profiles suggest a lack of established polymerase adaptation rather than the absence of future adaptive potential. Continued genomic surveillance and functional evaluation are required to determine whether these viruses can acquire mammalian-adaptive features following cross-species transmission.

Major mammalian-adaptive or virulence-associated substitutions in PB1, PA, and NP, including PB1-N105S/K577E, PA-T97I/S224P/A343S/D347E/S409N, and NP-N319K/T437M/K470R, were not detected in the Lao isolates (30, 31). Although Lao-associated patterns such as PB1-204T/222L, PA-142K/337A/418T, and NP-119I/432S/446R were observed, these are better interpreted as lineage- or region-associated variation rather than direct evidence of enhanced mammalian adaptation. The NA and MP segments also lacked major antiviral resistance markers, including NA-E119V/H275Y/N295S and M2-V27A/A30T/V/S31N/G34E (32). Because M2 reference sequences were unavailable, M2 variation was assessed only within the Lao dataset, where substitutions were observed at M2-12, M2-28, M2-42, M2-70, and M2-82. In NS1, Lao isolates retained conserved markers, including NS1-42S, NS1-92D, NS1-106M, NS1-149A, and the C-terminal ESEV PDZ-binding motif (33). The NS1-Δ80–84 deletion observed in one Vietnamese human reference sequence was absent from the Lao isolates and was therefore considered reference-specific (34).

Across the analyzed non-HA segments, Lao H5N1 clade 2.3.2.1e viruses did not show major known mammalian-adaptive or antiviral-resistance-associated substitutions. Instead, they exhibited distinct Lao-associated amino-acid patterns, including PB2-389R, PB1-204T/222L, PA-142K/337A/418T, NP-119I/432S/446R, NA-129A/I, NA-293H/Q, M2 internal variation, and NS1-207N. In contrast, HA marker analysis showed that the eight Lao avian H5 isolates retained a highly pathogenic HA backbone. All Lao isolates carried the polybasic HA cleavage motif PQRERRRKRGLF, which was identical to that observed in Vietnamese tiger isolates and most Cambodian human H5 sequences. Because polybasic HA cleavage sites are a major molecular determinant of systemic pathogenicity in H5 viruses, this conserved motif supports the highly pathogenic profile of the Lao isolates (35).

Several selected HA1 residues were located at receptor-binding-associated or RBS-proximal positions. Five Lao isolates carried HA1-94N, which was also observed in Vietnamese tiger, Vietnamese human, and Cambodian human sequences. This residue corresponds to a D94N-associated site previously linked to increased human-type α2,6 sialic acid receptor binding and enhanced HA-mediated fusion (36). All Lao isolates also retained HA1-154N and HA1-263T. The HA1-154N pattern was similar to Vietnamese human sequences but differed from Vietnamese tiger isolates carrying HA1-154D, suggesting HA marker diversity among regional H5 viruses rather than a standalone marker of transmissibility (37, 38). The conserved HA1-263T residue across Lao avian, Vietnamese tiger, Vietnamese human, and Cambodian human H5 sequences further indicates that this virulence-associated residue is shared among recent regional highly pathogenic H5 viruses (39, 40).

However, the Lao isolates lacked HA1-123P and HA1-497 K/R, which were detected only in selected human-derived sequences and have been reported to enhance α2,6 receptor binding in specific HA mutational backgrounds (16). Thus, although the Lao HA sequences showed variation at RBS-associated or RBS-proximal sites, they did not contain the complete HA marker pattern associated with enhanced human-type receptor binding. The substitutions at HA1-188 and HA1-223 should also be interpreted cautiously. Three Lao isolates carried HA1-188M and two carried HA1-223G, but these residues do not exactly correspond to previously validated human-adaptive substitutions such as T188I or Q/S223N/R/P (41–50).

The Lao avian H5 isolates shared a polybasic HA cleavage motif and selected HA marker residues with recent regional mammalian and human H5 sequences. However, the absence of HA1-123P and HA1-497 K/R, together with non-identical substitutions at HA1-188 and HA1-223, suggests RBS-associated HA variation rather than confirmed enhancement of human-type receptor binding. Further receptor-binding or phenotypic assays are needed to determine whether these sequence differences affect host receptor preference.

Structural modeling further distinguishes this study from previous Lao market surveillance reports. Whereas earlier studies documented AIV circulation and H5N1 clade 2.3.2.1e detection in market environments, our study integrated HA sequence variation with homology modeling and receptor-binding energy analysis. A/Hubei/1/2010 was used as a structural comparator because it is a human-infecting clade 2.3.2.1 H5N1 strain with an experimentally determined HA crystal structure available in the PDB (4KTH) (51). The representative Lao HA model showed high structural similarity to this human-infecting H5 template, with several variable residues located within or near the receptor-binding region. Although these findings do not demonstrate equivalent host range or infectivity, they provide a structural context for interpreting HA sequence variation in Lao isolates.

Recent structural and functional studies have shown that limited changes within or near the HA receptor-binding region can alter receptor preference or receptor-binding architecture in H5N1 viruses (17). However, receptor adaptation may remain incomplete even in mammalian-associated outbreaks, as shown for bovine-origin H5N1 HA, and HA receptor engagement can be further complicated by structural factors such as auto-glycan occupancy of the receptor-binding pocket (52, 53). Cambodian human H5N1 viruses have also been characterized using receptor-binding, pH-fusion, thermal stability, and antigenic analyses (54). In the present study, Lao isolates showed variation at HA1-94, HA1-188, and HA1-223, but lacked HA1-123P and HA1-497K/R, which have been associated with enhanced human-type receptor binding.

To predict functional changes associated with the observed HA mutations, in silico binding free energy evaluation was conducted. In nature, host sialic acid receptors exist as complex polysaccharides, making full-length physiological glycan modeling challenging. Furthermore, preliminary molecular docking trials with di- and tri-saccharides often generated poses that deviated from biologically relevant binding modes. Due to the abundance of flexible hydroxyl groups capable of forming promiscuous hydrogen bonds, automated docking scoring functions frequently caused the ligand to drift away from the core conserved receptor-binding site (RBS) residues, failing to reproduce known infection patterns. To resolve these docking-induced artifacts and capture the key linear and U-shaped topologies essential for host receptor recognition, multiple 3’-SLN and 6’-SLN trisaccharide conformations were extracted directly from verified crystal structures and structurally overlaid onto the target binding pocket (Figure 3; Supplementary Figure S2). To compensate for template limitations, side-chain flexibility within 6.0 Å was enabled during MM-GBSA minimization.

This aligned MM-GBSA framework successfully reflected the expected biological preferences: human-type 6’-SLN yielded lower predicted Δ*G*_bind_ values (−49.98 kcal/mol for A/Hubei/1/2010 and −51.40 kcal/mol for EPI_ISL_19,678,908) than avian-type 3’-SLN (−39.53 and −37.93 kcal/mol, respectively). The system reliability was further validated by the low-pathogenic avian control strain A/Duck/Singapore/3/1997 (1JSM), which correctly exhibited the opposite preference toward 3’-SLN (Supplementary Figure S2C). Nevertheless, as extensive molecular dynamics (MD) simulations were not conducted to assess long-term conformational stability, these thermodynamics predictions should be interpreted within the defined scope of this static-flexible modeling framework.

Therefore, these HA changes should be interpreted as RBS-associated sequence variations that warrant further structural and functional validation, rather than as confirmed markers of human-type receptor adaptation. Structural modeling of a representative Lao isolate supported this interpretation by locating several variable residues within or near the receptor-binding region. Consistent with previous studies demonstrating that a limited number of HA RBS substitutions can influence the recognition of α2,6-linked sialic acid receptors, MM-GBSA analysis predicted more favorable binding of the representative Lao HA model to the human-type α2,6-linked receptor analog than to the avian-type α2,3-linked analog (15–17). Notably, this predicted binding profile was observed despite the absence of PB2-627 K, a canonical marker of mammalian adaptation (9, 10, 55). This finding suggests that HA receptor-binding-related variation and PB2 mammalian-adaptive mutations may not necessarily emerge simultaneously during the early stages of zoonotic adaptation, consistent with recent assessments indicating that PB2 adaptations may arise during mammalian spillover while HA receptor switching remains incomplete or variable (56). In addition, comparative in silico analysis identified both conserved and strain-specific MHC class I-associated T-cell epitope regions within the HA proteins, suggesting potential differences in cellular immune recognition among the analyzed viruses. Nevertheless, molecular modeling, MM-GBSA calculations, and immunogenicity predictions are hypothesis-generating approaches and do not directly establish altered receptor specificity, enhanced infectivity in humans, or functional T-cell responses. Therefore, these computational findings require further validation through receptor-binding assays, cell-based infection studies, and functional immunological analyses.

The H5N1 viruses isolated from Lao PDR in this study highlight the potential importance of Lao live bird markets as regional settings for the continued circulation and diversification of clade 2.3.2.1e viruses. Whole-genome analysis showed that the Lao LBM-derived viruses belonged to clade 2.3.2.1e, were distributed across several phylogenetic sublineages, and possessed mosaic segment-level genomic backgrounds, suggesting that they are part of a broader regional evolutionary network rather than a single localized cluster. Although the Lao isolates had not acquired the canonical mammalian-adaptive PB2-E627K substitution, HA marker analysis combined with structural modeling and MM-GBSA receptor-binding analysis identified RBS-associated sequence and structural variation. These computational findings raise the possibility that some avian H5N1 viruses circulating in Lao market environments may possess receptor-binding-related features that could facilitate further mammalian adaptation if additional mutations are acquired. However, this study was based on only eight isolates collected during a relatively short period from 2023 to 2024, owing in part to limitations in sample availability under the collaborative surveillance and sample-sharing framework. Therefore, the present findings may not fully represent the genetic diversity, temporal trends, or epidemiological dynamics of H5N1 viruses circulating throughout Lao PDR and should be interpreted as preliminary. Nevertheless, the analysis of isolates obtained from multiple provinces over two consecutive years provides useful baseline information on the geographic and temporal diversity of clade 2.3.2.1e viruses in Laos. Continued systematic and longitudinal genomic surveillance using larger and more consistently collected sample sets, together with functional receptor-binding and infection experiments, will be required to validate these predictions and more accurately assess the zoonotic potential of clade 2.3.2.1e viruses circulating in Lao PDR and the wider Mekong region.

## Data Availability

The datasets presented in this study can be found in online repositories. The names of the repository/repositories and accession number(s) can be found in the article/Supplementary material.
