# Supplementary material for: H5N1 clade 2.3.2.1e lineage transition in Lao PDR during 2023–2024 associated with regional spread and human infection risk-associated receptor-binding features
Source: Front Public Health. 2026 Sep 3;14:1923867. doi: 10.3389/fpubh.2026.1923867 (PMC13582252; doi:10.3389/fpubh.2026.1923867)
Supplement: Supplementary file 1 [file Data_Sheet_1.docx]

Supplementary Material

# 1 Supplementary Figures and Tables

## 1.1 Supplementary Figures

**Supplementary Figure S1. Sequence alignment of the hemagglutinin (HA) of EPI_ISL_19678908 and the reference strain A/Hubei/1/2010 (H5N1, PDB ID: 4KTH).** Sequences were aligned using Clustal Omega and visualized with ESPript 3. Secondary structure elements derived from the A/Hubei/1/2010 crystal structure are annotated above the alignment, including α-helices and β-strands. Residues conserved between the two strains are highlighted with a red background, reflecting an overall sequence identity of 96%. Green numerals beneath the alignment indicate paired cysteine residues involved in disulfide bond formation.

**Supplementary Figure S2. Molecular interactions in the receptor-binding site with 3'-SLN.** Close-up view of the receptor-binding pocket of (A) A/Hubei/1/2010 (gray ribbon) and (B) EPI_ISL_19678908 (green ribbon) in complex with 3'-SLN (blue sticks). Key residues are shown as sticks and labeled. Yellow dashed lines indicate hydrogen bond interactions. (C) MM-GBSA-calculated binding free energies of HA complexes with 3′-SLN and 6′-SLN. More negative ΔG_bind values indicate more favorable predicted receptor binding. EPI_ISL_19678908 showed a more favorable binding energy with 6′-SLN than with 3′-SLN

**Supplementary Figure S3. Phylogenetic tree of PB2 gene.** The phylogenetic tree of PB2 gene from eight isolated H5N1 influenza virus was conducted using the neighbor-joining method implemented in MEGA X. The reliability of the tree topology was evaluated by bootstrap analysis with 1000 replicates.

**Supplementary Figure S4. Phylogenetic tree of PB1 gene.** The phylogenetic tree of PB1 gene from eight isolated H5N1 influenza virus was conducted using the neighbor-joining method implemented in MEGA X. The reliability of the tree topology was evaluated by bootstrap analysis with 1000 replicates.

**Supplementary Figure S5. Phylogenetic tree of PA gene.** The phylogenetic tree of PA gene from eight isolated H5N1 influenza virus was conducted using the neighbor-joining method implemented in MEGA X. The reliability of the tree topology was evaluated by bootstrap analysis with 1000 replicates.

**Supplementary Figure S6. Phylogenetic tree of HA gene.** The phylogenetic tree of HA gene from eight isolated H5N1 influenza virus was conducted using the neighbor-joining method implemented in MEGA X. The reliability of the tree topology was evaluated by bootstrap analysis with 1000 replicates.

**Supplementary Figure S7. Phylogenetic tree of NP gene.** The phylogenetic tree of NP gene from eight isolated H5N1 influenza virus was conducted using the neighbor-joining method implemented in MEGA X. The reliability of the tree topology was evaluated by bootstrap analysis with 1000 replicates.

**Supplementary Figure S8. Phylogenetic tree of NA gene.** The phylogenetic tree of NA gene from eight isolated H5N1 influenza virus was conducted using the neighbor-joining method implemented in MEGA X. The reliability of the tree topology was evaluated by bootstrap analysis with 1000 replicates.

**Supplementary Figure S9. Phylogenetic tree of MP gene.** The phylogenetic tree of MP gene from eight isolated H5N1 influenza virus was conducted using the neighbor-joining method implemented in MEGA X. The reliability of the tree topology was evaluated by bootstrap analysis with 1000 replicates.

**Supplementary Figure S10. Phylogenetic tree of NS gene.** The phylogenetic tree of NS gene from eight isolated H5N1 influenza virus was conducted using the neighbor-joining method implemented in MEGA X. The reliability of the tree topology was evaluated by bootstrap analysis with 1000 replicates.

**Supplementary Figure S11. Distribution of predicted T-cell epitope-associated regions across the HA protein sequence of A/Hubei/1/2010 (EPI_ISL_96896).** (A) Predicted MHC class I-associated regions identified using MHC-I binding, processing, and natural presentation tools. (B) Predicted MHC class II-associated regions identified using MHC-II binding, MHCII-NP, and CD4⁺ T-cell immunogenicity tools. Colored bars indicate the positions of predicted epitope-containing regions, and outlined boxes indicate regions supported by multiple prediction methods

**Supplementary Figure S12. Distribution of predicted T-cell epitope-associated regions across the HA protein sequence of A/duck/Vientiane/C2431/2023 (EPI_ISL_19678908)** (A) Predicted MHC class I-associated regions identified using MHC-I binding, processing, and natural presentation tools. (B) Predicted MHC class II-associated regions identified using MHC-II binding, MHCII-NP, and CD4⁺ T-cell immunogenicity tools. Colored bars indicate the positions of predicted epitope-containing regions, and outlined boxes indicate regions supported by multiple prediction methods

**Supplementary Table S1. Metadata of the 32 avian and environmental samples collected during active surveillance in Lao PDR between 2023 and 2024.** Bold indicates samples from which highly pathogenic avian influenza A(H5N1) virus was isolated.

| **No.** | **Sample ID** | **Collection date** | **Province** | **District** | **Sampling location** | **Host species/Source** | **Sample type** |
| --- | --- | --- | --- | --- | --- | --- | --- |
| 1 | M-U01987 | 8-Jun-2023 | Vientiane Capital | Xaythany | Danxang | Chicken | Oropharyngeal swab |
| **2** | **M-U02422** | **17-Jul-2023** | **Vientiane Capital** | **Xaythany** | **Danxang** | **Duck** | **Oropharyngeal swab** |
| **3** | **M-U02431** | **17-Jul-2023** | **Vientiane Capital** | **Xaythany** | **Danxang** | **Duck** | **Oropharyngeal swab** |
| 4 | M-U02929 | 15-Aug-2023 | Luangnamtha | Luang Namtha | Viengkham | Duck | Oropharyngeal swab |
| **5** | **M-U04369** | **15-Dec-2023** | **Vientiane Capital** | **Xaythany** | **Danxang** | **Duck** | **Oropharyngeal swab** |
| 6 | M-U04431 | 18-Dec-2023 | Oudomxay | Xay | Vienghai | Duck | Oropharyngeal swab |
| 7 | M-U04432 | 18-Dec-2023 | Oudomxay | Xay | Vienghai | Duck | Oropharyngeal swab |
| 8 | M-U04447 | 18-Dec-2023 | Oudomxay | Xay | Vienghai | Duck | Oropharyngeal swab |
| **9** | **M-UC85** | **23-Jan-2024** | **Vientiane Capital** | **Xaythany** | **Danxang** | **Duck** | **Oropharyngeal swab** |
| 10 | M-UC83 | 23-Jan-2024 | Vientiane Capital | Xaythany | Danxang | Duck | Oropharyngeal swab |
| 11 | M-UC106 | 23-Jan-2024 | Vientiane Capital | Xaythany | Danxang | Chicken | Oropharyngeal swab |
| 12 | M-UC111 | 23-Jan-2024 | Vientiane Capital | Xaythany | Danxang | Chicken | Oropharyngeal swab |
| **13** | **M-UC120** | **23-Jan-2024** | **Vientiane Capital** | **Xaythany** | **Danxang** | **Goose** | **Oropharyngeal swab** |
| 14 | M-UC124 | 23-Jan-2024 | Vientiane Capital | Xaythany | Danxang | Goose | Oropharyngeal swab |
| 15 | M-UC129 | 23-Jan-2024 | Vientiane Capital | Xaythany | Danxang | Environment | Fecal swab |
| 16 | M-UC625 | 20-Feb-2024 | Oudomxay | Xay | Vienghai | Duck | Oropharyngeal swab |
| 17 | M-UC627 | 20-Feb-2024 | Oudomxay | Xay | Vienghai | Duck | Oropharyngeal swab |
| 18 | M-UC629 | 20-Feb-2024 | Oudomxay | Xay | Vienghai | Duck | Oropharyngeal swab |
| 19 | M-UC630 | 20-Feb-2024 | Oudomxay | Xay | Vienghai | Duck | Oropharyngeal swab |
| 20 | M-UC630 | 20-Feb-2024 | Oudomxay | Xay | Vienghai | Duck | Oropharyngeal swab |
| **21** | **M-UC631** | **20-Feb-2024** | **Oudomxay** | **Xay** | **Vienghai** | **Duck** | **Oropharyngeal swab** |
| 22 | M-UC633 | 20-Feb-2024 | Oudomxay | Xay | Vienghai | Environment | Fecal swab |
| 23 | M-UC896 | 14-Mar-2024 | Vientiane Capital | Xaythany | Danxang | Duck | Oropharyngeal swab |
| 24 | M-UC897 | 14-Mar-2024 | Vientiane Capital | Xaythany | Danxang | Duck | Oropharyngeal swab |
| 25 | M-UC898 | 14-Mar-2024 | Vientiane Capital | Xaythany | Danxang | Duck | Oropharyngeal swab |
| 26 | M-UC1214 | 23-Apr-2024 | Vientiane Capital | Xaythany | Danxang | Chicken | Oropharyngeal swab |
| 27 | M-UC1853 | 16-May-2024 | Salavan | Salavan | Nalaek | Duck | Oropharyngeal swab |
| **28** | **M-UC2456** | **16-Jul-2024** | **Luangnamtha** | **Luang Namtha** | **Viengkham** | **Duck** | **Oropharyngeal swab** |
| 29 | M-UC3771 | 29-Oct-2024 | Bokeo | Houaixai | Pakhao Nuea | Duck | Oropharyngeal swab |
| **30** | **M-UC3773** | **29-Oct-2024** | **Bokeo** | **Houaixai** | **Pakhao Nuea** | **Duck** | **Oropharyngeal swab** |
| 31 | M-UC3786 | 29-Oct-2024 | Bokeo | Houaixai | Pakhao Nuea | Duck | Oropharyngeal swab |
| 32 | M-UC3818 | 29-Oct-2024 | Bokeo | Houaixai | Pakhao Nuea | Duck | Oropharyngeal swab |

**Supplementary Table S2. Comparison of selected PB2 amino-acid markers among H5N1 sequences.**

| **Contents** | **Accession/ID** | **PB2-271** | **PB2-389** | **PB2-627** | **PB2-655** |  |  |  |  |  |
| --- | --- | --- | --- | --- | --- | --- | --- | --- | --- | --- |
| **V**  **Tiger** | EPI_ISL_20081455 | T | R | K | V |  |  |  |  |  |
|  | EPI_ISL_19520689 | T | R | K | V |  |  |  |  |  |
|  | EPI_ISL_19520627 | T | R | K | V |  |  |  |  |  |
|  | EPI_ISL_19520622 | T | R | K | V |  |  |  |  |  |
|  | EPI_ISL_19520621 | T | R | K | V |  |  |  |  |  |
| **V human** | EPI_ISL_19031556 | T | R | K | V |  |  |  |  |  |
|  | EPI_ISL_19850663 | K | R | K | V |  |  |  |  |  |
| **C**  **human** | EPI_ISL_19312044 | T | R | K | V |  |  |  |  |  |
|  | EPI_ISL_18823967 | T | R | E | V |  |  |  |  |  |
|  | EPI_ISL_18939130 | T | R | K | V |  |  |  |  |  |
|  | EPI_ISL_18543642 | T | R | K | V |  |  |  |  |  |
| **L**  **avian** | **EPI_ISL_19678907** | **T** | **K** | **E** | **V** |  |  |  |  |  |
|  | **EPI_ISL_19678908** | **T** | **G** | **E** | **L** |  |  |  |  |  |
|  | **EPI_ISL_19678909** | **T** | **K** | **E** | **V** |  |  |  |  |  |
|  | **EPI_ISL_20050893** | **T** | **G** | **E** | **L** |  |  |  |  |  |
|  | **EPI_ISL_20459161** | **T** | **G** | **E** | **L** |  |  |  |  |  |
|  | **EPI_ISL_20459162** | **T** | **R** | **E** | **L** |  |  |  |  |  |
|  | **EPI_ISL_20459163** | **T** | **K** | **E** | **V** |  |  |  |  |  |
|  | **EPI_ISL_20459156** | **T** | **R** | **E** | **L** |  |  |  |  |  |

**Supplementary Table S3. Comparison of selected PB1 amino-acid markers among H5N1 sequences.**

| **Contents** | **Accession/ID** | **PB1-D3V** | **PB1-N105S** | **PB1-K577E** | **PB1-D622G** | **PB1-204** | **PB1-222** | **PB1-54** | **PB1-110** | **PB1-187** | **PB1-337** |
| --- | --- | --- | --- | --- | --- | --- | --- | --- | --- | --- | --- |
| **V**  **Tiger** | EPI_ISL_20081455 | V | N | K | G | I | M | K | T | R | L |
|  | EPI_ISL_19520689 | V | N | K | G | I | M | K | T | R | L |
|  | EPI_ISL_19520627 | V | N | K | G | I | M | K | T | R | L |
|  | EPI_ISL_19520622 | V | N | K | G | I | M | K | T | R | L |
|  | EPI_ISL_19520621 | V | N | K | G | I | M | K | T | R | L |
| **V human** | EPI_ISL_19031556 | ND | ND | ND | ND | ND | ND | ND | ND | ND | ND |
|  | EPI_ISL_19850663 | V | N | K | G | I | M | K | T | R | L |
| **C**  **human** | EPI_ISL_19312044 | V | N | K | G | I | M | K | T | R | L |
|  | EPI_ISL_18823967 | V | N | K | G | I | M | K | T | R | L |
|  | EPI_ISL_18939130 | V | N | K | G | I | M | K | T | R | L |
|  | EPI_ISL_18543642 | V | N | K | G | I | M | K | T | R | L |
| **L**  **avian** | **EPI_ISL_19678907** | **V** | **N** | **K** | **G** | **T** | **L** | **E** | **T** | **R** | **M** |
|  | **EPI_ISL_19678908** | **V** | **N** | **K** | **G** | **T** | **L** | **E** | **A** | **K** | **M** |
|  | **EPI_ISL_19678909** | **V** | **N** | **K** | **G** | **T** | **L** | **E** | **A** | **K** | **M** |
|  | **EPI_ISL_20050893** | **V** | **N** | **K** | **G** | **T** | **L** | **E** | **A** | **K** | **M** |
|  | **EPI_ISL_20459161** | **V** | **N** | **K** | **G** | **T** | **L** | **K** | **T** | **R** | **L** |
|  | **EPI_ISL_20459162** | **V** | **N** | **K** | **G** | **T** | **L** | **K** | **T** | **K** | **L** |
|  | **EPI_ISL_20459163** | **V** | **N** | **K** | **G** | **T** | **L** | **K** | **T** | **R** | **L** |
|  | **EPI_ISL_20459156** | **V** | **N** | **K** | **G** | **T** | **L** | **K** | **T** | **R** | **L** |

**Supplementary Table S4. Comparison of selected PA amino-acid markers among H5N1 sequences.** ND indicates that the corresponding PA amino-acid sequence was not available in the provided dataset and therefore could not be evaluated.

| **Contents** | **Accession/ID** | **PA-97 T97I** | **PA-224 S224P** | **PA-343 A343S** | **PA-347 D347E** | **PA-383 N383D** | **PA-409 S409N** | **PA-142** | **PA-337** | **PA-418** | **PA-210** |
| --- | --- | --- | --- | --- | --- | --- | --- | --- | --- | --- | --- |
| **V**  **Tiger** | EPI_ISL_20081455 | T | C | A | D | D | S | N | T | I | T |
|  | EPI_ISL_19520689 | T | S | A | D | D | S | N | T | I | T |
|  | EPI_ISL_19520627 | T | S | A | D | D | S | N | T | I | T |
|  | EPI_ISL_19520622 | T | S | A | D | D | S | N | T | I | T |
|  | EPI_ISL_19520621 | T | S | A | D | D | S | N | T | I | T |
| **V human** | EPI_ISL_19031556 | T | S | A | D | D | S | N | T | M | T |
|  | EPI_ISL_19850663 | T | S | A | D | D | S | N | T | I | T |
| **C**  **human** | EPI_ISL_19312044 | T | S | A | D | D | S | N | T | I | T |
|  | EPI_ISL_18823967 | T | S | A | D | D | S | N | T | I | T |
|  | EPI_ISL_18939130 | T | S | A | D | D | S | N | A | T | T |
|  | EPI_ISL_18543642 | T | S | A | D | D | S | N | T | I | T |
| **L**  **avian** | **EPI_ISL_19678907** | T | S | A | D | D | C | K | A | T | T |
|  | **EPI_ISL_19678908** | T | S | A | D | D | C | K | A | T | T |
|  | **EPI_ISL_19678909** | T | S | A | D | D | C | K | A | T | T |
|  | **EPI_ISL_20050893** | T | S | A | D | D | C | K | A | T | T |
|  | **EPI_ISL_20459161** | T | S | A | D | D | C | K | A | T | T |
|  | **EPI_ISL_20459162** | T | S | A | D | D | S | K | A | T | A |
|  | **EPI_ISL_20459163** | T | S | A | D | D | S | K | A | T | A |
|  | **EPI_ISL_20459156** | T | S | A | D | D | S | K | A | T | A |

**Supplementary Table S5. Comparison of selected NP amino-acid markers among H5N1 sequences.**

| **Contents** | **Accession/ID** | NP-105 M105V | NP-184 A184K | NP-319 N319K | NP-437 T437M | NP-470 K470R | NP-119 | NP-129 | NP-350 | NP-432 | NP-446 |
| --- | --- | --- | --- | --- | --- | --- | --- | --- | --- | --- | --- |
| **V**  **Tiger** | EPI_ISL_20081455 | V | K | N | T | K | M | A | T | G | K |
|  | EPI_ISL_19520689 | V | K | N | T | K | M | A | T | G | K |
|  | EPI_ISL_19520627 | V | K | N | T | K | M | A | T | G | K |
|  | EPI_ISL_19520622 | V | K | N | T | K | M | A | T | G | K |
|  | EPI_ISL_19520621 | V | K | N | T | K | M | A | T | G | K |
| **V human** | EPI_ISL_19031556 | V | K | N | T | K | I | A | T | S | R |
|  | EPI_ISL_19850663 | V | K | N | T | K | I | A | T | S | R |
| **C**  **human** | EPI_ISL_19312044 | V | K | N | T | K | I | A | T | S | R |
|  | EPI_ISL_18823967 | V | K | N | T | K | I | A | T | S | R |
|  | EPI_ISL_18939130 | V | K | N | T | K | I | A | T | S | R |
|  | EPI_ISL_18543642 | V | K | N | T | K | I | A | T | S | R |
| **L**  **avian** | **EPI_ISL_19678907** | **V** | **K** | **N** | **T** | **K** | **I** | **A** | **T** | **S** | **R** |
|  | **EPI_ISL_19678908** | **V** | **K** | **N** | **T** | **K** | **I** | **S** | **T** | **S** | **R** |
|  | **EPI_ISL_19678909** | **V** | **K** | **N** | **T** | **K** | **I** | **S** | **T** | **S** | **R** |
|  | **EPI_ISL_20050893** | **V** | **K** | **N** | **T** | **K** | **I** | **S** | **T** | **S** | **R** |
|  | **EPI_ISL_20459161** | **V** | **K** | **N** | **T** | **K** | **I** | **A** | **T** | **S** | **R** |
|  | **EPI_ISL_20459162** | **V** | **K** | **N** | **T** | **K** | **I** | **A** | **A** | **S** | **R** |
|  | **EPI_ISL_20459163** | **V** | **K** | **N** | **T** | **K** | **I** | **A** | **A** | **S** | **R** |
|  | **EPI_ISL_20459156** | **V** | **K** | **N** | **T** | **K** | **I** | **A** | **A** | **S** | **R** |

**Supplementary Table S6. Comparison of selected NA amino-acid markers among H5N1 sequences.**

| **Contents** | **Accession/ID** | **NA-99 E119V** | **NA-116 Q136K** | **NA-131 D151G/A** | **NA-132 R152K** | **NA-203 I223V/T/R/K** | **NA-255 H275Y** | **NA-275 N295S** | **NA-62** | **NA-129** | **NA-293** |
| --- | --- | --- | --- | --- | --- | --- | --- | --- | --- | --- | --- |
| **V**  **Tiger** | EPI_ISL_20081455 | E | Q | D | R | I | H | N | S | V | Q |
|  | EPI_ISL_19520689 | E | Q | D | R | I | H | N | S | V | Q |
|  | EPI_ISL_19520627 | E | Q | D | R | I | H | N | S | V | Q |
|  | EPI_ISL_19520622 | E | Q | D | R | I | H | N | S | V | Q |
|  | EPI_ISL_19520621 | E | Q | D | R | I | H | N | S | V | Q |
| **V human** | EPI_ISL_19031556 | E | Q | D | R | I | H | N | S | V | Q |
|  | EPI_ISL_19850663 | E | Q | D | R | I | H | N | S | V | Q |
| **C**  **human** | EPI_ISL_19312044 | E | Q | D | R | I | H | N | S | V | Q |
|  | EPI_ISL_18823967 | E | Q | D | R | I | H | N | S | V | Q |
|  | EPI_ISL_18939130 | E | Q | D | R | I | H | N | S | V | Q |
|  | EPI_ISL_18543642 | E | Q | D | R | I | H | N | S | V | Q |
| **L**  **avian** | **EPI_ISL_19678907** | E | Q | D | R | I | H | N | S | A | H |
|  | **EPI_ISL_19678908** | E | Q | D | R | I | H | N | S | A | H |
|  | **EPI_ISL_19678909** | E | Q | D | R | I | H | N | S | A | H |
|  | **EPI_ISL_20050893** | E | Q | D | R | I | H | N | S | A | H |
|  | **EPI_ISL_20459161** | E | Q | D | R | I | H | N | S | A | H |
|  | **EPI_ISL_20459162** | E | Q | D | R | I | H | N | S | V | Q |
|  | **EPI_ISL_20459163** | E | Q | D | R | I | H | N | S | V | Q |
|  | **EPI_ISL_20459156** | E | Q | D | R | I | H | N | S | V | Q |

**Supplementary Table S7. Comparison of selected MP amino-acid markers among H5N1 sequences.** ND denotes not determined because the corresponding M2 sequence was not provided and should not be interpreted as absence of mutation.

| **Contents** | **Accession/ID** | **M1-30 N30D** | **M1-43 I43M** | **M1-115** | **M1-215 T215A** | **M1-224** | **M2-12** | **M2-28** | **M2-42** | **M2-70** | **M2-82** |
| --- | --- | --- | --- | --- | --- | --- | --- | --- | --- | --- | --- |
| **V**  **Tiger** | EPI_ISL_20081455 | D | M | I | A | N | ND | ND | ND | ND | ND |
|  | EPI_ISL_19520689 | D | M | I | A | N | ND | ND | ND | ND | ND |
|  | EPI_ISL_19520627 | D | M | I | A | N | ND | ND | ND | ND | ND |
|  | EPI_ISL_19520622 | D | M | I | A | N | ND | ND | ND | ND | ND |
|  | EPI_ISL_19520621 | D | M | I | A | N | ND | ND | ND | ND | ND |
| **V human** | EPI_ISL_19031556 | D | M | V | A | S | ND | ND | ND | ND | ND |
|  | EPI_ISL_19850663 | D | M | V | A | S | ND | ND | ND | ND | ND |
| **C**  **human** | EPI_ISL_19312044 | D | M | V | A | S | ND | ND | ND | ND | ND |
|  | EPI_ISL_18823967 | D | M | V | A | S | ND | ND | ND | ND | ND |
|  | EPI_ISL_18939130 | D | M | V | A | S | ND | ND | ND | ND | ND |
|  | EPI_ISL_18543642 | D | M | I | A | S | ND | ND | ND | ND | ND |
| **L**  **avian** | **EPI_ISL_19678907** | D | M | V | A | N | K | T | I | D | S |
|  | **EPI_ISL_19678908** | D | M | V | A | S | K | T | I | E | S |
|  | **EPI_ISL_19678909** | D | M | V | A | S | K | T | I | E | S |
|  | **EPI_ISL_20050893** | D | M | V | A | S | K | T | I | E | S |
|  | **EPI_ISL_20459161** | D | M | V | A | S | K | T | I | D | N |
|  | **EPI_ISL_20459162** | D | M | V | A | S | K | I | V | E | S |
|  | **EPI_ISL_20459163** | D | M | V | A | S | R | I | I | E | S |
|  | **EPI_ISL_20459156** | D | M | V | A | S | K | I | I | E | S |

**Supplementary Table S8. Comparison of selected NS1 amino-acid markers among H5N1 sequences.**

| **Contents** | **Accession/ID** | **NS1-42 S42** | **NS1-80–84 deletion** | **NS1-92 D92E** | **NS1-103 F103S** | **NS1-106 M106I** | **NS1-138 F138L** | **NS1-149 A149V** | **NS1-207** | **NS1-215** | **NS1-227–230 PBM** |
| --- | --- | --- | --- | --- | --- | --- | --- | --- | --- | --- | --- |
| **V**  **Tiger** | EPI_ISL_20081455 | S | TITSV | D | F | M | L | A | D | S | ESEV |
|  | EPI_ISL_19520689 | S | TITSV | D | F | M | L | A | D | S | ESEV |
|  | EPI_ISL_19520627 | S | TITSV | D | F | M | L | A | D | S | ESEV |
|  | EPI_ISL_19520622 | S | TITSV | D | F | M | L | A | D | S | ESEV |
|  | EPI_ISL_19520621 | S | TITSV | D | F | M | L | A | D | S | ESEV |
| **V human** | EPI_ISL_19031556 | S | Δ80-84 | D | F | M | F | A | D | P | ESEV |
|  | EPI_ISL_19850663 | S | TITSV | D | F | M | F | A | D | S | ESEV |
| **C**  **human** | EPI_ISL_19312044 | S | TITSV | D | F | M | F | A | D | S | ESEV |
|  | EPI_ISL_18823967 | S | TITSV | D | F | M | F | A | D | S | ESEV |
|  | EPI_ISL_18939130 | S | TITSV | D | F | M | F | A | N | P | ESEV |
|  | EPI_ISL_18543642 | S | TITSV | D | F | M | F | A | D | S | ESEV |
| **L**  **avian** | **EPI_ISL_19678907** | S | TITSV | D | S | M | F | A | N | S | ESEV |
|  | **EPI_ISL_19678908** | S | TITSV | D | F | M | F | A | N | P | ESEV |
|  | **EPI_ISL_19678909** | S | TITSV | D | F | M | F | A | N | P | ESEV |
|  | **EPI_ISL_20050893** | S | TITSV | D | F | M | F | A | N | P | ESEV |
|  | **EPI_ISL_20459161** | S | TITSV | D | S | M | F | A | N | S | ESEV |
|  | **EPI_ISL_20459162** | S | TITSV | D | F | M | F | A | N | P | ESEV |
|  | **EPI_ISL_20459163** | S | TITSV | D | F | M | F | A | N | P | ESEV |
|  | **EPI_ISL_20459156** | S | TITSV | D | F | M | F | A | N | P | ESEV |

**Supplementary Table S9**. List of intermolecular interactions

| **HA** | **Host receptor** | **Interactions** | **HA amino acid residue** | **Distance (Å)** | **Mean ± s.d.** |
| --- | --- | --- | --- | --- | --- |
| A/Hubei/1/2010 (4KTH) | 3’-SLN | Hydrogen bond | TYR107 | 1.68 | 1.97 ± 0.29 |
|  |  |  | VAL147 | 1.82 |  |
|  |  |  | SER148 | 2.46 |  |
|  |  |  | ALA149 | 1.96 |  |
|  |  |  | ARG205 | 2.16 |  |
|  |  |  | GLN238 | 1.65 |  |
|  |  |  | GLN238 | 2.06 |  |
|  |  | Clashes | TYR107 | 1.86 | 2.32 ± 0.38 |
|  |  |  | TYR107 | 2.64 |  |
|  |  |  | HIS195 | 2.16 |  |
|  |  |  | GLN238 | 2.61 |  |
|  | 6’-SLN | Hydrogen bond | TYR107 | 1.71 | 1.99 ± 0.30 |
|  |  |  | VAL147 | 1.97 |  |
|  |  |  | SER148 | 2.28 |  |
|  |  |  | ALA149 | 2.12 |  |
|  |  |  | GLU202 | 2.43 |  |
|  |  |  | GLY237 | 1.84 |  |
|  |  |  | GLN238 | 1.61 |  |
|  |  | Clashes | TYR107 | 2.68 | 2.64 ± 0.06 |
|  |  |  | GLN238 | 2.59 |  |
| A/duck/Vientiane/C2431/2023 (EPI_ISL_19678908) | 3’-SLN | Hydrogen bond | TYR107 | 1.75 | 1.96 ± 0.21 |
|  |  |  | VAL147 | 1.76 |  |
|  |  |  | ALA149 | 2.00 |  |
|  |  |  | HIS195 | 2.18 |  |
|  |  |  | LYS205 | 2.27 |  |
|  |  |  | GLN238 | 1.76 |  |
|  |  |  | GLN238 | 2.03 |  |
|  |  | Clashes | GLU202 | 2.39 | 2.53 ± 0.20 |
|  |  |  | GLN238 | 2.67 |  |
|  | 6’-SLN | Hydrogen bond | TYR107 | 1.69 | 2.05 ± 0.35 |
|  |  |  | VAL147 | 2.17 |  |
|  |  |  | ALA149 | 2.11 |  |
|  |  |  | LYS234 | 2.25 |  |
|  |  |  | GLY237 | 1.79 |  |
|  |  |  | GLN238 | 1.59 |  |
|  |  |  | GLN238 | 2.69 |  |
|  |  |  | SER239 | 2.27 |  |
|  |  |  | GLY240 | 1.89 |  |
|  |  | Clashes | TYR107 | 2.64 | 2.61 ± 0.05 |
|  |  |  | GLN238 | 2.57 |  |
